# Progress in Medicine

**Published:** 1898-07-30

**Authors:** 


### Progress in Medicine.

### RENAL MEDICINE.

(*Concluded from page* 288.)

**Albuminuria.**—Dittmar[14] records the results of examination twice or oftener every day of 91 scarlatina patients at Glasgow during their entire stay in the hospital. Albuminuria or hæmaturia or both occurred in 52·7 per cent. of the cases, but dropsy or superficial œdema was noticed only in three of them, and a high tension pulse did not invariably accompany nephritis. He denies Mahomed's theory that a prealbuminuric stage exists with high tension and hæmaturia, which results in Bright's disease if unchecked, and considers that these cases of hæmaturia are merely transient forms of nephritis. Is there such a thing as hereditary albuminuria or renal weakness? Ranault[15] replies that the children of an eclamptic woman may show fatal albuminuria, that nephritis is found in the breast-fed children of nephritic patients, and that this disease may in other cases appear in them about the thirteenth year. He finds also instances where the disease appears in three generations, sometimes being cyclic in type. The latter is often the case in the offspring of gouty nephritics. The prognosis of hereditary albuminuria is, he thinks, bad.

H. Caiger[16] comments on the difficulty of valuing for insurance persons with cyclic albuminuria, as we know very little of the subsequent history of these cases, and how many finally succumb to nephritis. He refers to a patient where the albumen as usual disappeared during recumbency, though at times the amount was large, even exceeding one-third. The condition had existed for at least two and a half years with fairly good health, but with a marked history of neurotic tendencies in the patient and his family. Brandreth Symonds[17] points out that the most common fallacy is to mistake nucleo-albumen or mucin for true albumen. He checks all tests by Heller's, which he allows to stand for some hours in case of doubt. He does not even rely solely on heating and the addition of a few drops of nitric acid, unless confirmed by the cold nitric test. Though cyclic, adolescent, and fatigue albuminurias exist, he regards them all as pathological. Only when there is at last a continued absence of albumen from the urine in these cases can we give a good prognosis. A low specific gravity, the age above 40, a hard pulse, headaches, and abnormal body weight, are of bad omen. T. W. Bickerton agrees with him in looking on all forms as pathological, and states that out of 114 cases no less than 30 died in eight years; while Symonds adds that none of a certain group above 40 years. Over 40, in whom albumen was found, lived out their expected life.

**Toxicity of Urine.**—Pelligrini[18] has come to the conclusion that the amount of indican is a safe measure of the toxicity, and recommends Primavera's test. To carry out this he pours a drachm of urine into a tube, and adds slowly one-third of the volume of pure strong sulphuric acid and cools the mixture. About 20 drops of chloroform are then shaken up with it. This after settling takes a light blue tint if the indican is merely in normal amount. If the quantity present is greater the tint is darker. Auto-intoxication may thus be diagnosed, and an indication given for the need of intestinal antiseptics.

**Bacteriuria.**—The origin of bacteria in the water is not very clear. R. Barlow[19] analyses sixty-five cases where they were found, and denies their renal or urethral origin, referring them often to lesions of the intestines such as fissures of the anus. Among the species present were sarcinæ and B. coli, more rarely staphylococci and others. The urine is usually turbid and opalescent, but the reaction varies and a sediment falls containing most of the bacteria. A cure is not always easy to obtain, but salol internally and argonin or nitrate of silver locally are often useful. Van Ketel's[30] method of searching for tubercle bacilli when much pus is present has been recommended. One part of carbolic to twenty of the urine is shaken up for five minutes and allowed to settle. Cover glass specimens are dipped several times into alcohol and ether (equal parts) to render them transparent and are then stained in carbol fuchsin. If much blood is present it is almost useless to search for bacilli, but if necessary it is best to take a large number of slips and stain with anilin water fuchsin for 24 hours in an incubator. If the urine, however, is clean a small portion after settling may be centrifugalised and stained with carbol fuchsin at once. For the detection of lipuria, fatty casts, or chyluria Rieder[21] recommends the new stain, Sudan III., which colours the drops of fat a bright red or yellow colour. The concentrated stain in 96 per cent. alcohol is added to equal quantities of urine and similar alcohol, and the sediment examined under a microscope. Weaker alcohol is used afterwards to wash out any excess of the stain. Harris[22] has devised an instrument for obtaining urine from one ureter without the difficult process of cystoscopy. A septum is passed into the rectum or vagina which presses up the bladder in the middle line, forming a pond on each side, in which the urine collects, and can be drawn off by a special double-headed catheter dipping into either side. Guisy[23] reports a remarkable case of hysterical ischuria with vicarious elimination of urea from the nose, eyes, and other orifices. There was vomiting, but no diarrhœa or diaphoresis. Guisy himself saw the discharge from the nose and eyes, and had it tested, showing the presence of 3·6 grammes of urea to the litre. There appears no room for doubt as to the actual facts of the elimination of urea by these unusual channels, but the instance is certainly a very rare one.

**Alcaptonuria.**—Hirsch[24] noticed this excretion in a girl during an attack of intestinal catarrh. The urine was brownish-black, and became darker on standing, and, though acid, the addition of an alkali caused no deepening of tint as is usually the case. Fehling's solution was reduced on heating. There was no albumen or reaction with Nylander's bismuth test, and the conditions passed away in four days. This substance is often mistaken for sugar, but there is no polarisation, nor does fermentation take place with yeast. A very full account of the peculiarity and of the recorded cases is given by Futcher, who does not regard it as a disease, for it often exists throughout life without other symptoms. The phenylhydrazine test will also show the absence of sugar, but the substance, possibly homogentisinic acid, gives a blue colour with ferric chloride, and reduces nitrate of silver in ammonia.

**Peptone.**—In testing for this substance, E. Freund[25] recommends that albuminous urine should be acidified, boiled, and then made neutral. To 10 cc. of this, while unfiltered, he adds three drops of a 10 per cent. solution of lead acetate. Next, after filtering, the biuret test may be employed without risk of error, and will show one part of peptone in 12,000. It is perhaps more correct to speak of this substance in urine as a propeptone or albumose, though a true peptone is found in putrefying albuminous urine. Cattaneo[26] finds that the so-called peptone is very common in the infectious diseases of children, but is of no value for prognosis or diagnosis. It is often seen when suppuration or bacterial action is present. W. H. Thompson[27] remarks that injections of peptones cause an increased secretion of dilute urine so great that the total amount of urea and nitrogen is also increased. Some of the peptone is retained in the body.

**Albumose** has been found in some six cases where sarcoma of the bones of the trunk existed, but how the malignant growth leads to this complication is not clear. Rosin[28] reports an interesting case of the kind where there was sarcoma, but no nephritis. T. R. Bradshaw[29] describes another where some undetermined disease of the vetebræ existed, and where the albumose was precipitated spontaneously. Coagulation took place with nitric acid, and the coagulum dissolved on heating. Other instances have been observed where ovarian tumours or pneumonia without sarcoma were present. Hæmatoporphyrin has been discussed by Ogden,[30] who reports an instance after an attack of diphtheria in a woman who had long suffered from rheumatism and a mitral lesion. Vomiting and diarrhœa and loss of sensation up to the waist were observed, and two doses of trional were given for insomnia, after which the reddish-purple urine was passed. It will be remembered that it has several times been seen after sulphonal, as well as in plumbism, tuberculosis, and intestinal hæmorrhage.

**Treatment.**—An interesting paper on cystinuria, with accounts of two cases, is given by Walter G. Smith.[31] Both patients were in good health, though one suffered from rheumatism, and in both the cystin crystals were transitory. It appears to be a product of disordered metabolism, and is related to lactic acid fermentation. Smith considers it has no relation to uric acid or rheumatism, and that, like diaminuria, it may possibly be due to intestinal micro-organisms. Hence he recommends the use of internal antiseptics. Knowsley Sibley[32] has found the Tallerman hot air baths produce extraordinary relief in asthmatic bronchitis associated with severe albuminuria. In one case the arm only was placed in the superheated air from time to time; profuse sweating took place, and both the lungs and the kidneys improved rapidly. L. Guthrie questions the use of hot baths in kidney disease, because the perspiration is increased without a corresponding elimination of urea; but Sibley replies that these localised baths increase both the quantity of urine, that of urea, and still more that of uric acid excreted. Urotropin[33] has given good results in renal colic, phosphaturia, pyelitis, and cystitis. Casper gives as much as a drachm daily in the latter cases with good success, though much smaller doses have been recommended by others for fear of hæmaturia. In old persons with pyelitis and enlarged prostate it seems especially valuable. In acute nephritis with œdema, Liégeois[34] prescribes tannin in daily doses of one or two grammes, with a milk diet; and in the subacute stage he adds theobromine with brilliant results. If cardiac failure and dilatation follow, he then gives digitalis, but never where there is hypertrophy and high tension pulse.

[14] Glasgow Med. Jour., Dec. [15] Brit. Med. Jour., Jan. 1. [16] South African Med. Jour., Jan. [17] Med. Rec., Jan. 29. [18] Brit. Med. Jour., Feb. 5. [19] Amer. Jour. Med. Sci., Mar. [20] Brit. Med. Jour., May 7. [21] Amer. Jour. Med Sci., May. [22] Jour. Amer. Med. Assoc., Jan. 29. [23] Lancet, Feb. 19. [24] Amer. Jour. Med. Sci., Feb. [25] Brit. Med. Jour., Mar. 19. [26] Les Nouv. Remédes, April 8. [27] Brit. Med Jour., Mar. 12. [28] Amer. Jour. Med. Sci., Feb. [29] Lancet, April 30. [30] Amer. Jour. Med. Sci., April. [31] Practitioner, May. [32] Clin. Jour., April 6. [33] Amer. Jour. Med. Sci., April. [34] New York Med. Jour., Dec. 11.

